# Twin pregnancy with one normal fetus and one complete hydatidiform mole: Outcome of expectant management

**DOI:** 10.12669/pjms.40.2(ICON).8950

**Published:** 2024-01

**Authors:** Samia Shuja, Sadia Rauf

**Affiliations:** 1 Samia Shuja, FCPS. Professor, Department of Obstetrics and Gynecology, Indus Hospital & Healthcare Network (IHHN), Sheikh Saeed Memorial Campus (SSMC), Karachi, Pakistan; 2 Sadia Rauf, FCPS. Senior Registrar, Department of Obstetrics and Gynecology, Indus Hospital & Healthcare Network (IHHN), Sheikh Saeed Memorial Campus (SSMC), Karachi, Pakistan

**Keywords:** Twin pregnancy, Complete mole, Normal fetus, Gestational trophoblastic neoplasia, Live birth

## Abstract

Twin pregnancy with one fetus and one complete mole falls amongst extremely rare obstetric situations. The spectrum of complications associated with it is wide. Once diagnosed, the choice between continuation and termination of pregnancy depends upon couple’s preference and readiness to accept possible complications. Key to success lies in clinical vigilance and tailoring management according to emerging needs during pregnancy and follow up. We present a case of twin pregnancy with complete hydatidiform mole and coexisting normal fetus diagnosed at 25 weeks. Pregnancy was complicated by anemia, gestational diabetes mellitus, recurrent vaginal bleeding, intrauterine growth restriction and iatrogenic preterm delivery. She was followed for development of GTN, none occurred.

## INTRODUCTION

The combination of one complete mole and one normal fetus in a twin pregnancy is rare and complex clinical entity.^1^ The dilemma associated with decision of termination vs continuation of pregnancy poses a challenge to the obstetrician. Live birth rate of 40% and insignificant increase in risk of gestational trophoblastic neoplasia are enough to support expectant management.^2^ Here we present one such case with good maternal and fetal outcomes.

## CASE REPORT

**Fig.1:**
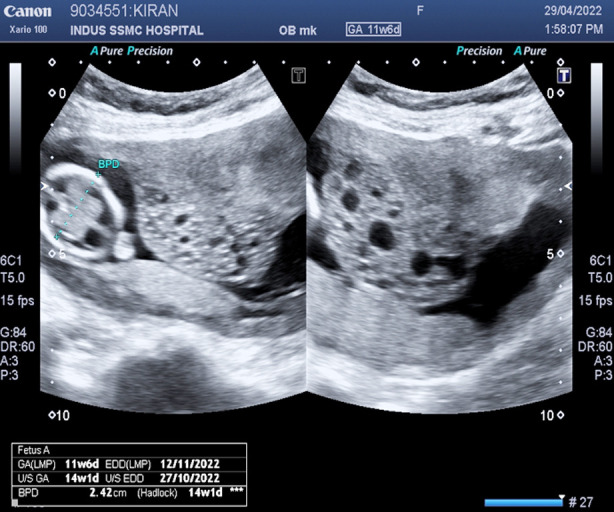
Ultrasound at 11+6 weeks showing fetus with normal placenta and complete hydatidiform mole.

Twenty-eight years old, G_5_P_2_^+2^, with history of previous one caesarean section booked for antenatal care at ten weeks and four days of gestation. She had two alive children, last-born being two years and five months old. Both miscarriages occurred in first trimester and last miscarriage was reported to be three years ago. Index pregnancy was spontaneous and planned. Dating scan corresponded with period of amenorrhea. Other than pallor and suprapubic scar, examination was unremarkable. Booking investigations revealed haemoglobin of 7g/dl and fasting blood glucose of 95mg/dl. Anemia and gestational diabetes mellitus were diagnosed and managed as per unit protocols. At 14 weeks, she reported in ER with moderate bleeding per vaginum. Ultrasound was done to check fetal viability. In addition to intrauterine pregnancy corresponding to 14 weeks and one day alive, ultrasound revealed an iso-echoic lesion measuring 8.5 X 8.1 cm with multiple tiny cystic spaces, in anterior wall of uterus (Fig.1).

**Fig.2:**
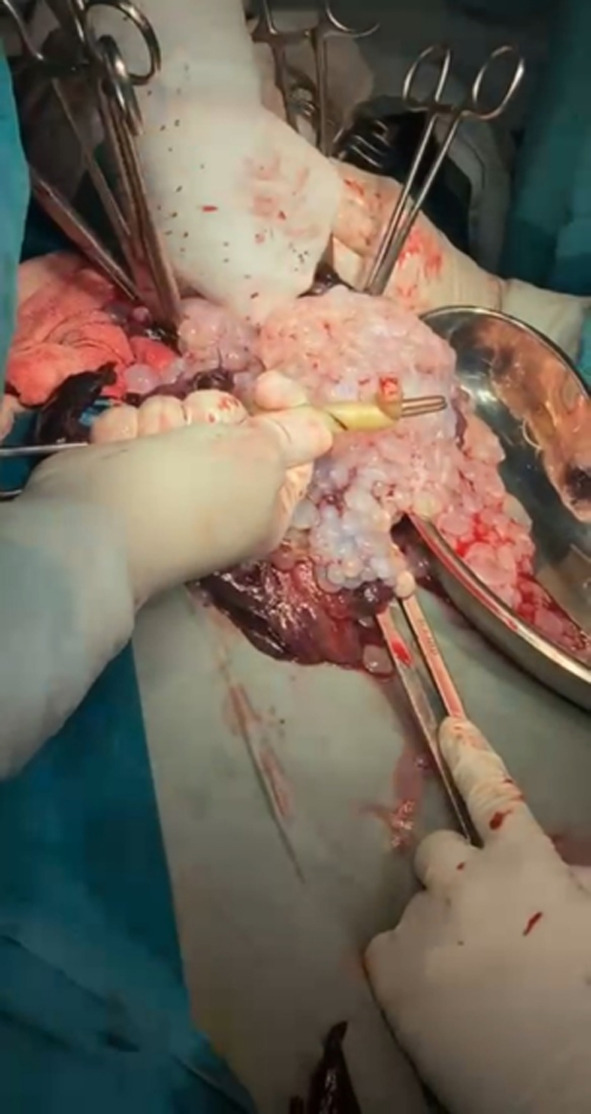
Molar tissue escaping along with placenta of live baby.

This raised suspicion of a coexisting molar pregnancy. The suspicious lesion was followed with serial scans. At follow up scan, done after two weeks, possibility of sub mucosal fibroid with cystic changes was also considered as one of the differentials. The same suspicion was maintained at the time of anatomy scan. At third scan, the “lesion” had grown and sonologist confidently labelled it as twin pregnancy with complete mole in one sac and normal fetus and placenta in the other. Fetal growth parameters corresponded with gestational age and placenta was in upper segment. In view of very high-risk pregnancy, couple was counselled. Options of termination vs continuation of pregnancy were discussed focusing on associated risks and benefits. The couple opted for continuation. Patient was seen by senior consultant on every antenatal visit. She was admitted to hospital at 14, 25 and 27 weeks of gestation with history of bleeding that used to settle on its own. Her last admission to hospital was at 30 weeks. With all preparations of a preterm high-risk caesarean section, pregnancy was carried to 32 weeks when an episode of moderate bleeding necessitated delivery. From one sac, an alive, active baby girl, weighing 1.4 Kg was delivered as breech. Along with expulsion of normal looking placenta and membranes, one litre of typical molar tissue followed (Fig.2). Uterus was doubtlessly empty at closure. Molar tissue was sent for histopathological examination.

Routine postoperative care was offered, and breast feeding was encouraged. Recovery was uneventful. Beta HCG level on first postoperative day was 117291.21 IU/L and dropped to 501.78 IU/L after two weeks. Histopathology report confirmed normal placenta of live baby and complete hydatidiform mole in apparently molar tissue. Patient was placed on standard follow up along with barrier contraception.

Further decline in beta HCG level was rather slow and below the cut-off on 100th day of delivery. Irregular minimal bleeding continued for 12 weeks. Regular menstruation achieved thereafter.

## DISCUSSION

The rare phenomenon of complete hydatidiform mole coexisting with a viable live fetus (CMCF) was reported for the first time in 1914.^3^ It is believed to occur at highly variable rates; 1/22,000–1/100,000 pregnancies globally.^4^ Factors responsible for wide variations in reported prevalence may include local epidemiological factors and accuracy of diagnosis. In view of increased use of assisted reproductive techniques (ART) globally, the likely hood of increase in incidence of such pregnancies is high.^5^ Affected mother is at risk of complications of multiple pregnancy coupled with that of complete mole. Hence, refractory vaginal bleeding, thyrotoxicosis, trophoblastic emboli, hyperemesis, preeclampsia and gestational trophoblastic neoplasia are recognized complications. Molar placenta previa/accreta and placental abscess are rare complications. Threats to fetus include spontaneous abortion, growth restriction, preterm birth and intra-uterine fetal demise.^5^ Reported maternal death rate is 1.4%.^6^ Live birth rate is around 40%.^2^ In this case, repeated episodes of vaginal bleeding led to multiple hospital admissions and necessitated iatrogenic preterm birth.

Ultrasound remains the mainstay of diagnosis. MRI can be used as an alternative imaging tool for cases posing diagnostic challenge. The other two conditions that need to be excluded are: singleton pregnancy consisting of a partial mole and one viable fetus; and a combination of partial mole with a twin in one amniotic sac, and one normal twin in the other.^7^ The importance of correct diagnosis lies in the fact that the fetus in complete hydatidiform mole and coexisting fetus (CHMCF) has a chance to survive contrary to the fetus of partial mole who tends to die.^1^ In our case sonographic picture became clear at 25 weeks of pregnancy when molar tissue had grown significantly.

The choice between continuation of pregnancy with close supervision and elective termination, depends upon couple’s preference after a detailed counselling session.^4^ Regarding Beta HCG estimation, recommendations and practices are varied; some suggest measuring Beta HCG during pregnancy while others support first measurement at delivery followed by weekly tests and plotting values on a standard regression curve adjusted for local reference standards.^2^,^8^ Level of beta HCG at the time of diagnosis is considered as one of the predictors of pregnancy outcome.^4^ Considering that management of such cases is mainly dependent on clinical course and couple’s wishes, we did first Beta HCG estimation after delivery thus saving the cost.

Other biochemical markers such as alpha fetoprotein (AFP) and pregnancy associated plasma protein A(PAPP-A) may be helpful in establishing diagnosis during pregnancy where imaging is not helpful.^7^

## CONCLUSION

Twin pregnancies with one fetus and one complete mole, despite being highly complex, can be managed expectantly with favorable outcome. Other than couple’s motivation and compliance, clinician’s willingness to take up the challenge can make a difference. Support of a well-equipped system offering reliable imaging and laboratory facility, blood bank and adult and neonatal intensive care units are the basic requirements to deal with such cases.

### Authors’ Contribution:

**SS** did literature search, writing & final approval of manuscript.

**SR** did data collection, literature search, drafting & editing of manuscript.
